# Supplementary material for: Are carbon emissions trading and green financial instruments synergistic? -Comprehensive quantitative research based on content analysis
Source: PLoS One. 2024 Mar 7;19(3):e0298601. doi: 10.1371/journal.pone.0298601 (PMC10919601; doi:10.1371/journal.pone.0298601)
Supplement: S1 Data — (ZIP) [file pone.0298601.s001.zip › Data/地级市Z_F工作报告.zip!/╣π╬≈/╖└│╟╕█╩╨/└┤╫╘╣1⁄2╓┌║┼í╛┬φ┐╦╩2╛▌═°í┐_2013.docx]

各位代表：

现在，我代表市人民政府向大会作工作报告，请予审议，并请市政协委员和列席会议的同志提出意见。

2012年工作回顾

过去一年，在自治区党委、政府和市委的坚强领导下，我们紧紧围绕“定格局、干大事、掀高潮、全面上”的主基调，以迎接建市20周年“干成20件大事，大干20件大事”为重点，凝心聚力，克难攻坚，推动经济社会加快发展，各项事业取得新成效，较好地完成了市五届人大二次会议确定的目标任务。

一、经济发展保持良好态势，“双二十”大事全面推进

在国内外形势复杂多变，全球经济增长放缓等诸多困难的情况下，全市上下抢抓机遇，积极应对，实现了经济持续平稳较快发展。

——多项指标位居全区前列。全市生产总值457.5亿元，增长12.5%；财政收入52.4亿元，增长18.1%；规模以上工业实现增加值199亿元，增长19.2%；港口货物吞吐量1.01亿吨，增长11.5%。人均地区生产总值、农民人均纯收入、港口货物吞吐量、外贸进口总额、海关税收、工业经济效益综合指数、更新改造投资增速等7项重要经济指标排全区第一。工业增加值、财政收入、房地产开发投资、社会消费品零售总额、城镇居民人均可支配收入等17项经济指标总量或增幅位居全区前列。

——兴起“双二十”大事建设热潮。积极谋划，推动实施关系全局、影响长远的“双二十”大事，产业、城建、文化旅游、民生等重点领域的项目扎实推进，全年实施“双二十”项目218个，完成投资量占全社会固定资产投资的85%。

——实现具有里程碑意义的重大突破。防城港钢铁基地项目获国家批准开工建设，东兴国家重点开发开放试验区（以下简称“东兴试验区”）实施方案获国务院批准，亿吨大港如期建成，奠定了我市迈入全国沿海开放城市第一集团的发展格局。钦崇高速上思段建成通车，结束了十万大山不通高速公路的历史；南防高铁全线贯通并试运营，我市即将进入高铁时代。

二、东兴试验区建设全面启动，开放合作取得新进展

先行先试扎实推进。争取自治区出台了支持试验区的优惠政策。高起点、高标准启动试验区建设总体规划、土地利用总体规划等18项规划编制工作。东兴国门口岸改造、国际旅游集散中心等项目建成使用；北仑河国际商贸城等一批重点项目开工建设。区内边境旅游办证业务等8项先行先试政策全面实施。中国人民银行东兴市支行挂牌成立，争取国开行、建行等10多家银行的融资支持。全国重点开发开放试验区建设工作现场会在我市召开，对东兴试验区工作给予充分肯定。

对外开放深入拓展。成功举办中越边境（东兴—芒街）商贸旅游博览会、中国—东盟博览会东兴试验区专题推介会、金滩沿边开发开放合作论坛。开展国际交流活动，与波兰热舒夫市结为友好城市。对外贸易继续保持快速增长，外贸进出口总额49亿美元，增长19.1%；边贸成交额突破200亿元，增长24.3%。

三、产业集群加快打造，工业经济提速增效

千百亿产业加快形成。三大项目建设共完成投资99亿元，钢铁基地项目正式开工建设，金川铜镍项目全面推进，红沙核电项目进入设备安装阶段。企沙工业区完成工业项目投资107亿元，在全区115个产业园区中排名第一。上思朗姆酒试产成功，成为自治区重点扶持千亿元产业的重要基地。惠禹油菜籽加工项目建成投产，全市粮油产业实现产值272.7亿元，成为全国第一大食用油籽加工基地。盛隆冶金通过技术改造，实现产值120亿元。金源镍合金、信润石化等项目开工建设。中电防城港电厂二期工程启动，神华煤电一体化工程前期工作加快推进，企沙东岛现代能源化工基地启动规划。全市工业总产值达到790.5亿元，产值100亿元企业2家、10亿元企业10家，亿元企业82家。

工业效益再创新高。规模以上工业经济效益综合指数达617.3%，连续6年稳居全区第一。企业创新能力不断提高，高新技术企业从1家增至7家，高新技术产业产值突破百亿元，达130亿元。强力推进冶金、建材、造纸、制糖、磷化工等重点行业的节能减排。落实环境倒逼机制，开展环境安全风险隐患大排查，依法关停5家企业。

四、交通设施不断完善，国际枢纽加快形成

建成亿吨大港。港口基础设施建设加强，18#~22#泊位技改工程基本完成，403#~407#泊位进入后方陆域吹填和设备安装阶段；防城港钢铁基地专用泊位、云约江公用码头加快建设，企沙西港区逐步形成。港航服务体系不断完善，广西第一艘巴拿马级远洋船舶“北部湾光辉”号从我市首航，全球第二大集装箱航运公司——地中海航运公司在我市开通集装箱外贸航线，外贸集装箱航线和班轮居全区之首。港口吞吐量实现历史性突破，进入亿吨大港行列。

交通物流加速发展。交通运输能力不断提升，防东高速公路完成路基工程。完成铁路货运量3167万吨，占广西沿海总运量的七成。物流园区建设加快，建成全国最大的煤炭进口储存配送基地，北部湾大货车物流服务中心完成一期建设，投资10亿元的东盟农产品国际采购物流园项目落户。

五、城市功能不断增强，海湾城市魅力彰显

新区建设步伐加快。全年完成城建投资302亿元。中心区、桃花湾北部片区路网基本形成，西湾环海大道建设扎实推进，文昌大道东段开工建设。中央商务区建设加快。市青少年活动中心、文化艺术中心投入使用，市科技馆、图书馆、博物馆进入布展阶段。防城港收费站新站、城市候机楼建成启用。学校、公园、市场等公共服务设施建设加快推进。城市综合交通枢纽、中心区集贸市场开工建设。成立土地城建综合执法指挥中心，拆除违章建筑13.5万平方米。

海湾城市品位提升。北部湾海洋文化公园、仙人山公园、白鹭公园等项目基本建成，北部湾大道等景观绿化亮化成效显著，西湾城市沙滩一期建成使用，西湾海域整治顺利推进。房地产保持快速发展态势，累计完成房地产开发投资130亿元，商品房销售面积145万平方米，居全区第5位，27个20万平方米以上大型房地产项目建设全面铺开，房地产十强企业恒大地产集团入驻。成功创建广西园林城市，获“南珠杯”竞赛特等奖，城市人居环境明显改善。

六、旅游商贸日趋兴旺，现代服务业加快发展

旅游业蓬勃发展。全年游客量和旅游收入分别同比增长19.4% 、29.7%。屏峰雨林公园、白浪滩景区成功创建4A级景区，冲皇沟、瑶山大峡谷漂流等一批景区改造升级。江山半岛旅游度假区获得“中国最美休闲度假旅游胜地”称号。珍珠湾国际旅游区和十万大山山地休闲旅游规划开展编制。防城港至越南下龙湾海上航线恢复通航。新增宾馆酒店80家。成功举办江山半岛国际欢乐季、城建旅游大篷车巡游等大型宣传推介活动，成为重要的自驾游目的地。

商贸业繁荣兴旺。桂海东盟商汇、月亮岛购物广场、中心区建材家居广场等大型购物中心建成使用。引进北京华联超市、苏宁电器等大型零售企业和连锁品牌。农村商贸活跃，“家电下乡”销售额1.5亿元。消费市场繁荣兴旺，社会消费品零售总额增速跃居全区第3位。

现代服务业加速兴起。新增各类金融机构11家；资本市场发展迈出新步伐，23家企业进入自治区上市培育备案库；金融机构人民币贷款余额275亿元，增长24.3％，增幅居全区第一。文化娱乐、体育健身、家政、养生等现代服务业加快发展。

七、农村经济转型提速，农业基础逐步夯实

农村经济稳步发展。全市实现农林牧渔业总产值100亿元，粮食产量实现“四连增”。林下经济产值突破10亿元。建成120个“万元村屯”、331个“万元片区”、263户“十万元示范农户”。农民人均纯收入达7539元，连续五年全区排名第一。

农业产业化步伐加快。甘蔗、“红金系列”等优势产业形成规模。深水抗风浪网箱养殖、小水体流水养鱼、中药材种植等特色产业快速发展。新增市级农业产业化龙头企业5家，新成立农民专业合作社42家，直接带动8.2万农户从事产业化经营。发展生态休闲农业，新建农家乐、渔家乐示范点13个。开展科技特派员创业行动，一批农业新技术、新品种得到推广，广西农业标准化生产技术示范基地从2家增至6家。

农村基础设施不断改善。投资4.35亿元，实施小型农田水利、海河堤防建设和病险水库除险加固、高效节水灌溉工程。投入1.7亿元，新建农村道路307公里。完成“六统强基工程”、村级“十六有”阵地建设，在全区率先开展乡镇“五化十有”规范化建设。

八、民生福祉显著改善，社会事业全面发展

民生投入进一步加大。民生支出占全市财政支出比重达71%。保障性住房在全区率先完成开竣工任务。城乡就业、城乡低保、农村五保供养、孤儿供养工作全区领先。投入12.2亿元，全面实施教育振兴工程。广西财经学院防城港校区建成招生，实现了我市全日制高校零的突破。新建乡镇幼儿园4所，桃源小学、北港小学、金海湾小学建成使用，市第四中学开工建设。投入4.99亿元，积极发展医药卫生事业，扎实推进医药卫生体制改革，在全区率先实行新农合融入金融功能“一卡通”。人口计生“幸福家园”品牌全区推广。为59万城乡居民建立电子健康档案。全面实施新一轮扶贫攻坚战，全年农村脱贫人口2.5万人。

文化等各项事业快速发展。京族民歌、上思舞鹿、防城采茶戏等入选自治区级非物质文化遗产名录。获评“中华诗词之市”。农家书屋实现村村全覆盖，红沙网箱养殖协会农家书屋成为全国示范点。国际海上龙舟节、京族文化节、金花茶节、森林旅游节等重大节庆档次进一步提升，“欢乐西湾”、“明江之夜”广场文化活动形成品牌。举办全市首届全民健身运动会。首家数字电影院“世纪星城”正式营业。新增847个自然村通广播电视。稳步推进生态市建设工作，获批成为第四批国家可再生能源建筑应用示范城市，白鹭志愿者保护协会成立。林改等各项改革深入推进。专利申请达335件，增速居全区第一。

社会保持和谐稳定。信访维稳工作扎实推进，全市信访量下降14.6%。抗灾减灾、应急处置能力增强，成功抗击“启德”、“山神”台风和其他灾害。未发生重特大安全生产事故。全市社会治安稳定，专项严打整治斗争深入开展，北仑河沿线打私“断链”行动取得显著成效。社会公众安全感全区排位从第七跃升至第三。

一年来，外事、侨务、统计、审计、国资监管、工商、税务、海关、检验检疫、海事、通信、邮政、供销、地震、人防、消防、保密、气象、决策咨询、社会科学、地方志、档案、宗教、残疾人、妇女儿童等工作取得新进展。国防教育和国防后备力量建设深入开展，连续三次荣获“全国双拥模范城”。边海防基础设施建设进一步加强。全市民族团结，边疆巩固安宁，百姓安居乐业。

九、政府建设力度加大，服务能力不断提高

深入开展“学政策促发展”活动，强化学习型政府建设。依法行政进一步加强。扎实开展扩权强县工作，市级下放县级行政审批事项529项。完善绩效考评办法，开展民主评议科长活动。严格执行集体议事规则和决策程序，年内审议政府重大决策事项90多个；重视决策咨询工作，政府决策更加科学化。建立完善产业发展、城市建设指挥部等“6+2”工作机制，创新项目建设管理模式。坚持项目集中联合审批制度，进一步提高行政审批效率。实施“公寓+商铺”新型安置政策，争取低丘缓坡荒滩等未利用地开发利用试点，开展批而未用闲置土地清理专项行动，盘活存量土地1.55万亩。加大财政投资评审和政府投资项目审计力度，核减不合理资金8.65亿元。强化融资，政府性银行贷款管理进一步加强；积极偿还政府债务，有效化解财政风险。自觉接受人大、政协监督，广泛听取意见，办理人大代表建议27件、政协委员提案158件，答复率均为100%。

各位代表！

过去一年，我市在艰难形势下经济保持平稳较快发展，社会各项事业不断进步，成绩来之不易。这是自治区党委、政府和市委正确领导的结果，是市人大、市政协有效监督、大力支持的结果，是中直、区直单位和驻军驻警支持帮助的结果，是各级各部门和全市人民团结拼搏、真抓实干的结果。在此，我谨代表市人民政府，向全市广大干部群众，向解放军指战员、武警官兵和公安民警，向各民主党派、工商联、无党派人士、人民团体，向所有关心、支持、参与我市现代化建设的各界人士和海内外朋友，表示衷心的感谢和崇高的敬意！

在肯定成绩的同时，我们也清醒地看到，受国际国内经济放缓的影响，我市经济社会发展仍然面临较多困难和问题。主要是：钢铁基地项目尚未大规模投资建设，一批我市已投入大量配套资金的重大产业项目还没有形成效益；第三产业发展滞后；城区功能不够健全；征地拆迁安置工作难度加大；贫困山区农民增收困难；资金、人才、港口集疏运等瓶颈制约亟待破解；一些群众反映的难点问题还没有得到很好解决。对此，我们要千方百计加以克服和改进，努力把政府工作做得更好。

2013年工作安排

2013年，是贯彻落实党的十八大精神的开局之年，是“十二五”规划两步走战略第一步目标的实现之年，是建市20周年的喜庆之年。根据市委的统一部署，今年政府工作的总体要求是：深入贯彻落实党的十八大精神，按照“稳中求进”的总基调，围绕“在全区率先发展、在沿海后来居上、迈入沿海开放城市第一集团”的要求，干成大事献厚礼，突破重点上台阶，圆满完成“十二五”规划第一步奋斗目标，以辉煌成就向建市20周年献礼。

今年我市经济社会发展预期目标为：地区生产总值增长13%，财政收入增长13%，全社会固定资产投资增长23%，全部工业总产值增长30%，社会消费品零售总额增长17%，外贸进出口总额增长15%，实际利用外资增长18%，港口货物吞吐量增长9.4%，城镇居民人均可支配收入增长14%，农民人均纯收入增长15%，居民消费价格涨幅控制在5%以下，城镇登记失业率控制在3.8%以内，人口自然增长率控制在10‰以内，万元地区生产总值能耗和主要污染物排放量控制在自治区要求范围内，各项社会事业取得新成效。

重点抓好以下工作：

一、全力推动工业经济上新台阶，加快建设现代化临海工业基地

强化工业主导地位，重点实施“226工程”，加快培育千百亿元产业，打造千亿元产业园区，力争工业产值超千亿元。

加快打造钢铁、有色金属两大千亿元产业。钢铁项目主厂区要实质性开工建设，建成指挥部大楼和两个配套码头，加快推进厂区道路、护厂河等项目。金川项目要加快供电线路和“三路一管”建设，加快铁路专用线修建和敏感区征地拆迁工作，确保40万吨铜冶炼项目上半年投产。盛隆技改一期项目实现投产，完成产值200亿元。

加快打造企沙、大西南两大千亿元园区。加强企沙、大西南两大园区道路、供水、供电、污水处理、标准厂房及生活配套设施建设。企沙工业区重点加快“三路两桥”、云约江南作业区1#~4#泊位工程等项目建设，加快建设云约铁路站；强化有色金属、钢铁上下游产业招商。大西南临港工业园加快工业品专业市场和盛弘冶金项目建设，开工建设综合建材市场，加快渔洲坪磷酸企业搬迁。同时，加快企沙东岛现代能源化工基地规划建设。加快茅岭、上思工业园和东兴红木文化产业园建设。注重培育扶持民营企业，引进就业导向型项目，增强产业发展活力。

加快推进六个超百亿元产业项目。抓好大海粮油、枫叶粮油等企业技改，年内粮油产值力争达到300亿元；建成产值超100亿元的金源镍合金项目；尽快建成产值超400亿元的信润石化项目；开工建设产值超100亿元的海森特装备制造项目；开工建设产值超100亿元的科元新材料项目；推进红沙核电主厂区及应急道路、输出工程建设，抓好中电防城港电厂二期项目及配套码头建设，尽快形成产值超100亿元的电力产业。实施“抓大壮小扶微”工程，推进朗姆酒、汇圣40万吨啤酒等一批项目。延伸大项目产业链，发展一批上下游产业，形成大企业“顶天立地”、小企业“铺天盖地”的局面。

加快推进产业转型升级。强化创新驱动，深入实施发明专利倍增计划，加大科技型企业扶持培育力度。加强产学研合作，推动磷酸技术标准研究，加快朗姆酒工程研究院、盛隆冶金新材料研究中心、金花茶工程技术研究中心等重点研发机构建设，提高我市科技创新能力。建设循环产业园，提高资源综合利用率。深入实施环境倒逼机制，做好节能减排工作；加快建设节能综合配套项目，加强内部能耗管理；充分利用地热、光伏能源，强化公共节能；大力发展精深加工和循环经济。

二、奋力开创东兴试验区建设新局面，全面提升开发开放水平

全面落实试验区建设实施方案，拓展先行先试领域，努力把东兴试验区建成全国沿边领先、广西最高水平的开放平台，积极探索沿边开放新模式，为我市跨越发展注入新的动力。

深化先行先试。进一步解放思想，敢闯敢试。以先行先试政策为突破口，全面推进更多领域的创新。用足用活用好国家和自治区支持试验区的政策，制定相应配套措施，争取多方支持。建立完善协调配合、分工明确、高效运转的工作机制。积极申请扩大口岸开放范围，在出入境和通关便利化上实现新突破。实施聚才扬帆、白鹭英才计划，在人才特区建设上迈出大步伐。加大金融创新力度，筹建东兴银行，加快东投集团企业债券发行工作。创新融资模式，拓宽融资渠道。创新园区发展方式，实行便捷的准入审批制度，强化招商引资，使东兴试验区成为产业聚集的新高地。

搭建开放平台。要以国际经贸区为重点，全面推进试验区五大功能区建设。一是搭建边境贸易发展平台。着力提升东兴边民互市贸易区功能，建成景观苗木进口基地，推进水果、畜禽等产品进口口岸的规划及申报工作；加快建设北仑河国际商贸城，打造国际旅游集散中心品牌商业街区，形成集商贸、旅游、加工为一体的边贸中心。二是搭建跨境合作平台。加快中越（东兴—芒街）跨境经济合作区路网、安置区等建设，推进杨屋互市贸易点改扩建工程并尽快验收投入使用，争取北仑河二桥口岸区开工建设，积极吸引产业进驻。三是搭建特殊开放平台。在国际经贸区积极申报实施高度开放的海关特殊监管政策，重点推进金滩旅游岛复岛工程、江山半岛国际旅游项目。四是搭建综合保税平台。加快综合保税区选址和申报工作，推动国际航运、保税物流、加工贸易等产业发展。

构建国际通道枢纽。建成使用403#~407#泊位，完成18#~22#泊位技改，加快云约江1#泊位及一批产业配套码头建设；开工建设513#~516#泊位、旅游码头等项目。加快企沙东、企沙南、茅岭等港区规划编制工作。启动企沙东港区航道等项目前期工作，力争企沙南作业区开工建设。进一步完善港口集疏运。加快城市综合交通枢纽建设，建成高铁客运站、防东高速公路。加快钦防高速公路改扩建工程、滨海公路防城港段、防城至江山一级路、港口至东兴一级路改造、马路至垌中二级公路等项目建设；推进企沙至茅岭公路、吴圩机场经上思至江山高速公路、防城至东兴高速铁路等项目前期工作。

拓展对外交流合作。全面深化与越南等东盟国家的全方位交流合作，加大与港澳台、珠三角、大西南等周边地区的合作与交流。开展“走进试验区”大型招商推介活动及“金滩论坛”系列活动。用好中国—东盟博览会等平台，提升中越边境（东兴—芒街）商贸旅游博览会品牌。突出“招大引强”，着力引进世界500强和国内500强企业，切实提高签约项目兑现率。吸引设立更多中外合资、合作企业。鼓励更多企业“走出去”。

三、积极推进新型城镇化，加快建设生态海湾城市

按照新型城镇化要求，以城市新区建设为重点，着力完善功能配套，提升城市品质，加快打造边海明珠、美丽港城。

加快完善城市功能。重点抓好中心区、桃花湾等新区建设。实施“聚人工程”，健全水、电、气、公共交通等市政设施建设，完善教育、医疗、休闲娱乐等公共服务设施配套，加紧布局一批学校、幼儿园、医院、超市、银行、市场。合理开发利用城市地下空间，科学布局地下管网。推进城南新区、西湾新城等新城区基础设施建设，加快建设江山大道西段及长途汽车客运站，东湾大道、西湾环海大道、针鱼岭大桥、文昌大道东段建成通车。建成伏波文化公园、仙人山公园、龙马—明珠公园、防城区人民广场。推动公车新城与中心区的联动发展。继续实施绿化亮化景观提升工程，加快打造西湾景观，完成城市沙滩二期项目。按照15分钟生活圈规划建设公共休闲服务配套设施，方便市民生活。

增强县城承载能力。加快县城基础设施和公共服务设施建设，推动产业、人口和资源向县城聚集。上思县城要抓好“一江两岸”城区的开发建设，突出发展生态旅游、商贸、休闲养生等产业，打造北部湾休闲后花园。东兴市要推动城区东拓北扩，规划建设江平新城，突出发展边境贸易、进出口资源加工、旅游等产业，打造全国一流的边境口岸城市。

推进乡镇建设试点。加快重点镇建设，在企沙、那良等综合条件好的中心镇进行试点，创新投融资管理体制机制，通过政府引导、市场推动，加强城镇基础设施建设，培育壮大特色支柱产业，探索具有防城港特色的乡镇投资开发模式。

提升城市管理水平。加快完成城市总体规划和试验区建设总体规划的修编和报批，抓紧编制重点区域、重要地段的城市设计。加强城市管理，着重整治沿海、沿路、城乡结合部的乱搭乱建现象，加大违章建筑打击力度。扎实开展城乡清洁工程，解决建设工地、城中村、乡镇所在地的脏乱差问题。下大力气抓好安置区建设，努力破解征地搬迁难题。优先发展公共交通，规范出租车经营行为。完善市、区两级联动机制，实施市容环境综合考评。具备条件的城关镇尽快完成撤镇设街道。稳步推进撤乡设镇工作。

建设美丽防城港。全面落实主体功能区规划，加强十万大山、防城江上游等生态功能区保护，切实保护海洋环境。强化东湾、西湾海域治理，整治港区粉尘污染，加快城区、集镇、园区垃圾污水处理设施建设。实施高速公路、一级路沿线、城市周边风貌改造和林象改造工程。开工建设江山半岛绿道、慢行系统。实施造林绿化工程。积极推进创建国家园林城市和自治区卫生城市工作。加快国家可再生能源建筑应用示范城市建设。

四、加快产业结构调整，推动第三产业大发展

必须把发展第三产业作为优化产业结构的战略重点，采取强力措施，加大扶持力度，切实改变第三产业发展滞后的现状，增强经济发展活力。

提升旅游业。加快珍珠湾国际旅游区、十万大山旅游区、红沙群岛等规划建设。推进环西湾景区、金滩旅游岛、白浪滩等景区建设，争创江山半岛5A级景区。利用富集的滨海旅游资源，打造红沙至竹山三岛三湾滨海旅游精品线路；利用“中国长寿之乡”、“中国氧都”品牌，打造穿越十万大山休闲养生旅游线路；积极争取国家通过边境旅游异地办证验收，打造跨境旅游线路。突出特色，丰富内涵，打响节庆文化旅游品牌。出台导游、旅行社奖励办法，加强旅游营销推介。加快建成北港皇冠、德城、渭博等星级酒店，加快大环坞、江山半岛国际旅游集散中心建设，建设一批上档次、有特色的旅游购物及娱乐场所。

做旺商贸业。着力打造世界风情步行街、防城区人民路、中央商务区、东兴口岸区、红木商街等核心商业区。中心区集贸市场年内建成使用。加快推进北部湾商业中心、桃花湾集贸市场、沙潭江汽车贸易园、东盟农产品国际采购物流园等项目建设。实施“万村千乡市场工程”，完善农副产品平价商店、标准化菜市场等商业网点。

壮大物流业。以大港口、大工业为依托，大力发展现代物流业，加快物流园区建设，打造区域性国际物流中心。东湾物流加工产业园要加快中外运仓储物流基地、北部湾大货车物流服务中心、安派达矿产品仓储物流配送中心等项目建设，确保年内竣工运营。加快公车物流园区、冲仑物流园区、茅岭矿业物流园建设，配套完善基础设施，推进北部湾现代物流中心等项目建设，着力引进一批实力强、规模大的企业入园。完善集装箱等航运业扶持政策，积极引进大型船运公司、知名物流运营商，开辟新航线，提升国际中转能力，拓展国际物流发展空间。

做强房地产业。引导和支持房地产业健康发展，重点抓好21个30万平方米以上的房地产项目建设。强化全海景生态海湾城市品牌，提高房地产开发质量，完善公共服务配套，规范小区物业管理，打造一批高品质商住区。加大宣传推介营销力度，吸引更多外来人口前来置产创业。

发展现代服务业。继续实施“引金入港”战略，鼓励新型金融机构发展，拓展人民币跨境结算业务，推进资本市场建设，支持多元化融资，加快培育企业上市。培育发展创意机构。推动信息咨询、保险、船代货代、法律服务、会计等中介服务业发展。搭建服务业发展平台，扶持中小服务企业发展。

五、进一步夯实三农基础，全面增强农村发展活力

按照保供增收惠民生、改革创新添活力的目标，加大农村改革力度、政策扶持力度、科技驱动力度，加快社会主义新农村建设，推动城乡一体化，进一步强化农业、惠及农村、富裕农民。

大力发展现代农业。在稳定粮食生产的同时，着力发展优势特色农业，发展“红金系列”、食用菌、中药材等优质高效产业。整合农村土地，积极发展农民专业合作社，实施规模经营，壮大蔗糖、林下养鸡等产业。充分利用海洋资源优势，发展深水网箱养殖、浅海滩涂养殖、虾塘养殖、远洋捕捞和海产品加工。继续抓好“菜篮子”工程。加快农业综合开发和特色基地建设，培育壮大农业龙头企业，推动金花茶、金鲳鱼等产业化开发。大力发展农副产品加工业，建设一批休闲农业旅游村（点），推进农业向“接二（产）连三（产）”延伸。加强农产品物流体系建设，推动农超对接、农校对接、农企对接等多种形式的产销衔接。

加强农村基础设施建设。推动农村公共交通、电力、供水、通讯升级改造，加快城乡基础设施一体化进程。抓好小型农田水利、农村饮水安全工程，加快大垌水库建设，完善防洪抗旱排涝体系。加强村屯道路建设，解决边远地区行路难和农产品输出难问题。继续推进乡镇“五化十有”规范化建设。扎实推进率先基本实现气象现代化试点工作，提高气象服务能力。深入推进新一轮兴边富民行动，改善边境地区、少数民族聚居点生产生活条件。

六、实施居民收入倍增计划，切实提高人民生活水平

千方百计增加居民收入，促进经济增长与收入增长同步，实现城镇居民人均可支配收入超25000元、农民人均纯收入超8600元。

着力增加农民收入。一是增加家庭经营收入。重点发展优势特色种养业和农副产品加工业，实施“农户万元增收工程”，建设“万元村屯”300个、“万元片区”17万亩和“示范农户”320户，以现代农业促进农民增收。实施“千万林农千元增收”工程，大力发展林下经济。引导边民从事边贸致富。二是增加工资性收入。加强就业培训，扩大就业，增加农民务工收入，年内实现农村新增劳动力转移就业1.8万人。积极扶持微小企业发展，鼓励务工农民回乡创业。三是增加转移性收入。落实中央和自治区各项强农惠农富农政策，做好财政配套支持，增加生产补贴收入。深入推进新一轮扶贫攻坚，做好机关及企事业单位定点扶贫和对口支援工作，突出产业扶贫，带动6200户脱贫。四是提高农民财产性收入。发展多种形式的适度规模经营，让更多农民获得财产性收入。鼓励农民采取土地经营权、资金、产权入股等方式建立农村合作经济组织，通过壮大农村集体资产增加收入。

着力增加城镇居民收入。一是进一步提高企事业单位职工工资收入。健全完善企业工资分配机制、工资正常增长机制，按照自治区部署再调整最低工资标准，增长15%以上。完善事业单位收入增长机制。二是切实增加居民经营性收入。全面落实国家和自治区鼓励民间投资和民营经济发展的一系列政策措施，促进全民创业、全民增收。大力发展微型企业，新增微型企业700户，新增就业7000人。新增个体工商户1300户。三是实施更加积极的就业政策。发展劳动密集型产业和服务业，积极开发公益性岗位。加大对特殊困难群体的就业援助力度，确保“零就业家庭”至少有1人就业。全年完成城镇新增就业2.38万人。

七、积极发展各项社会事业，提升社会管理水平

统筹经济社会协调发展，加大投入力度，促进公共服务均等化，不断满足人民群众需求。

坚持优先发展教育。基本完成学前教育三年行动计划。推进义务教育均衡发展。加快全市中小学布局调整和校舍标准化改造，切实改善农村学校寄宿条件，进一步解决城区义务教育“大班额”问题。提升高中办学水平。深化职业教育攻坚，启动理工职业学校建设，完善市职业教育中心建设，进一步办好广西财经学院防城港校区，加快建设市行政学院（市委党校）新校区。

提升全民健康水平。深化医药卫生体制改革，抓好县级公立医院综合改革。市精神病院和港口区人民医院建成使用，开工建设市儿童医院。完善农村三级卫生服务网络，发展社区卫生事业。全面推行新农合融入金融功能“一卡通”工作。实现人口计生“幸福家园”工程村村全覆盖。继续实施防治艾滋病攻坚工程、母婴健康“一免二补”幸福工程等项目。建成三光企老干部活动中心。建设市食品药品、农产品质量安全检测中心，提高监管服务能力。

推动文化大繁荣。大力弘扬“敬海敬山敬人，开放开明开拓”的港城精神，提高市民素质。精心策划，办好建市20周年系列活动。推进公共文化体系建设，完成市博物馆、科技馆、图书馆及国防教育基地陈列展示并对外开放。加强文化艺术精品创作，推进“梦幻北部湾”项目，精心创编音乐剧《过桥风吹》，加快文化遗产保护和利用。推动文化体育进校园、进社区、进单位，不断丰富群众文体生活。着力壮大文化产业，搭建面向东盟的文化交流合作平台。

加强和创新社会管理。健全新型社区管理和服务体制，提高城乡基层组织社会管理服务能力。抓好十万山华侨林场的改制工作。加强各种事故隐患大排查大整治，坚决遏制各类重特大安全事故。加强应急处置工作，预防群体性事件的发生。推进社会管理信息化建设。加强国防动员、国防后备力量建设和人民防空工作，推进双拥共建活动。创新信访接访工作机制，维护群众合法权益。积极推进平安法治防城港建设，加强社会治安综合治理，加大扫黄打非和社会治安重点地区排查整治力度，扎实推进边海防综合管控设施项目规划建设，严厉打击重特大犯罪活动，保持打击走私的高压态势，不断提高人民群众的安全感和满意度。

八、着力为民办实事，打造幸福和谐防城港

坚持以人为本，高度关注民生，将更多财力向民生倾斜，继续办好惠民实事，让群众得到更多实惠。今年，我们将积极筹措资金，集中力量实施十项为民办实事工程：

1.社保惠民工程。投入1030万元，对陆地边境线0—20公里范围内17.1万农民参加新农合进行补助，每人财政扶持60元。巩固提高城乡居民社会保险参保率，健全城乡最低生活保障制度，加大弱势困难群体救助帮扶力度。进一步提高社保待遇，城镇职工和城镇居民基本医疗保险统筹基金报销比例提高5%，提高职工大病救助医疗待遇水平，降低城镇居民住院医疗费用报销起付标准。

2.安居惠民工程。投入2.4亿元，抓好保障性安居工程建设，新开工1928套（户），竣工2900套（户），入住2740户；廉租住房补贴300户；改造农村危房4148户。

3.健康惠民工程。投入1150万元，为城镇参保职工5万人免费体检，为符合条件的夫妇免费孕前优生体检。

4.教育惠民工程。投入3.58亿元，新建、改扩建一批中小学，新建、续建一批乡镇幼儿园，继续实施农村义务教育学生营养改善计划、困难学生资助项目。

5.交通惠民工程。投入6700万元，修建农村硬化道路200公里，实现全市村村通硬化道路。

6.土地整治惠民工程。投入4500万元，整治耕地1000公顷。

7.生态惠民工程。投入4100万元，修建一批垃圾中转站和公厕，在上思县开展村屯环境连片整治。

8.文化惠民工程。投入1100万元，在40个社区建设一批书屋、篮球场等文体设施，实施村级公共服务中心项目和图书馆、文化馆（站）等公共文化场所免费开放项目，继续实施社区、农村公益电影放映工程。

9.水利惠民工程。投入4亿元，开展农田水利、饮水安全、病险水库除险加固、海河堤防等水利基础设施建设。

10.社区服务惠民工程。投入5700万元，建成水营体育综合馆，继续推进完善社区卫生服务中心建设，对28条小街小巷进行改造。

九、切实转变工作作风，打造务实清廉政府

严格落实中央关于改进工作作风、密切联系群众的八项规定，转变作风，真抓实干，切实提高政府服务水平。

着力加强作风建设。精文简会，简化接待。轻车简从，深入一线，切实做到悉民情、解民忧。实行流程再造，减少审批环节。健全“马上办”制度，以雷厉风行、一抓到底的作风，特事特办、急事急办，提高办事效率。

着力推进依法行政。严格按照法定权限和程序行使权力，建设法治政府。完善政务服务中心综合服务功能，推进政务公开。自觉接受人大、政协监督，认真办理人大代表建议和政协委员提案。健全重大决策咨询机制，广泛听取社会各界意见，提高政府决策水平。加强税收征管和政府债务管理，强化投资评审和审计监督，提高理财水平。

着力加强廉政建设。认真落实党风廉政建设责任制，严格遵守廉洁从政各项规定。严惩贪污腐败。加强工程建设等领域专项整治，决不允许各级领导干部违规干预工程项目、干预招投标。厉行勤俭节约，反对铺张浪费，严格控制“三公”经费，全力营造风清气正的政务环境!

各位代表！

“雄关漫道真如铁，而今迈步从头越”。防城港市已经站在新的历史起点上，发展前景更加美好。让我们紧密团结在以习近平同志为总书记的党中央周围，以党的十八大精神为指引，在市委的坚强领导下，万众一心，开拓奋进，以辉煌成就向建市20周年献礼，为率先全面建成小康社会而努力奋斗！
